# Characterizing human lung tissue microbiota and its relationship to epidemiological and clinical features

**DOI:** 10.1186/s13059-016-1021-1

**Published:** 2016-07-28

**Authors:** Guoqin Yu, Mitchell H. Gail, Dario Consonni, Michele Carugno, Michael Humphrys, Angela C. Pesatori, Neil E. Caporaso, James J. Goedert, Jacques Ravel, Maria Teresa Landi

**Affiliations:** 1 Genetic Epidemiology Branch, Division of Cancer Epidemiology and Genetics, National Cancer Institute, NIH, DHHS, Bethesda, MD USA; 2 Biostatistics Branch, Division of Cancer Epidemiology and Genetics, National Cancer Institute, NIH, DHHS, Bethesda, MD USA; 3 Infections and Immunoepidemiology Branch, Division of Cancer Epidemiology and Genetics, National Cancer Institute, NIH, DHHS, Bethesda, MD USA; 4 Epidemiology Unit, Fondazione IRCCS Ca’ Granda - Ospedale Maggiore Policlinico, Milan, Italy; 5 Department of Clinical Sciences and Community Health, University of Milan, Milan, Italy; 6 Institute for Genome Sciences, University of Maryland School of Medicine, Baltimore, MD USA

**Keywords:** Air pollution, Tumor stage, 16S rRNA

## Abstract

**Background:**

The human lung tissue microbiota remains largely uncharacterized, although a number of studies based on airway samples suggest the existence of a viable human lung microbiota. Here we characterized the taxonomic and derived functional profiles of lung microbiota in 165 non-malignant lung tissue samples from cancer patients.

**Results:**

We show that the lung microbiota is distinct from the microbial communities in oral, nasal, stool, skin, and vagina, with *Proteobacteria* as the dominant phylum (60 %). Microbiota taxonomic alpha diversity increases with environmental exposures, such as air particulates, residence in low to high population density areas, and pack-years of tobacco smoking and decreases in subjects with history of chronic bronchitis. Genus *Thermus* is more abundant in tissue from advanced stage (IIIB, IV) patients, while *Legionella* is higher in patients who develop metastases. Moreover, the non-malignant lung tissues have higher microbiota alpha diversity than the paired tumors.

**Conclusions:**

Our results provide insights into the human lung microbiota composition and function and their link to human lifestyle and clinical outcomes. Studies among subjects without lung cancer are needed to confirm our findings.

**Electronic supplementary material:**

The online version of this article (doi:10.1186/s13059-016-1021-1) contains supplementary material, which is available to authorized users.

## Background

The human body harbors extraordinarily diverse communities of microbes (microbiota) that are increasingly thought to be crucial for human health. Recent studies have revealed intriguing correlations between specific patterns of human microbiota and various diseases, including autoimmune disorders, diabetes, obesity, and even psychiatric conditions [1–6].

The healthy human lung was traditionally considered sterile. However, since the first culture-independent report of microbiota in asthmatic airways [7], more than 30 studies using diverse molecular techniques have suggested that the healthy human lung is also home to bacteria (reviewed in [8, 9]). Because lung biopsy collection is not ethical in healthy human subjects, the study of lung microbiota has been mostly based on bronchoalveolar lavage (BAL), bronchoscopic brushing, or sputum samples. Reliance on these samples to determine lung microbiota is problematic due to contamination by the upper respiratory tract or oral microbiota [8]. To date, only four studies on human lung tissue microbiota have been published. These studies have considerable limitations, including small sample size (*n* < 33) and use of samples mostly from patients with severe lung diseases, such as chronic obstructive pulmonary disease (COPD) or cystic fibrosis [10–13]. Therefore, the characteristics of lung tissue microbiota remain largely unknown.

In this study, we characterized the taxonomic and derived functional profiles of the microbiota in non-malignant lung tissue samples from 165 lung cancer patients and compared them with previously published profiles from other body sites (including oral cavity, nasal cavity, gut, skin, and vagina from the Human Microbiome Project [14]). We also evaluated the associations between features of non-malignant lung microbiota and epidemiological and clinical characteristics. Finally, we compared non-malignant with tumor lung microbiota.

## Results

### Characteristics of the study participants

A total of 165 non-malignant lung tissue samples which generated at least 1000 sequence reads per sample (mean ± standard deviation (sd), 4091 ± 4167) were included in the analysis. Additional file 1: Table S1 describes the study population. The participants were mainly males (83 %) with a median age of 66.6 years; 53 % lived in the urban area of Milan (see Additional file 2: Figure S1 for a map of studied residential areas); most were smokers (51 % current and 43 % former) with a median of 45.3 pack-years and 43.5 years of smoking; 10 to 25 % self-reported a history of bronchitis, emphysema, and pneumonia; based on spirometry, 45 % subjects had a history of COPD. Most had tumor in the upper or lower lobe and 3 % had tumor in the principal bronchus; 38 % had squamous cell carcinoma and 59 % had adenocarcinoma; 92 % were diagnosed in stages IA, IB, IIA, IIB, and IIIA and only 8 % had more advanced cancer stages (IIIB, IV), as expected, since patients with later cancer stages are usually treated with systemic therapy instead of surgery. No patients had received chemotherapy, radiation therapy, or other treatments for lung cancer before surgery. The patients survived a median of 201.9 weeks after lung cancer diagnosis.

### Taxonomic and functional profiles of the non-malignant lung tissue microbiota

**Fig. 1:**
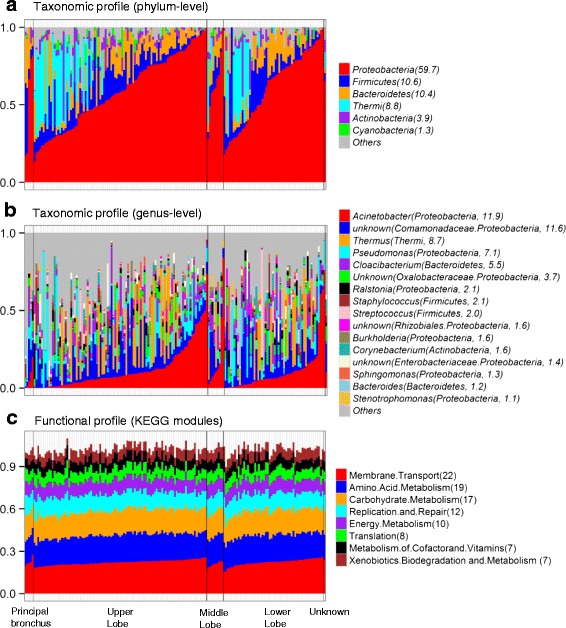
Taxonomic and functional profiles of non-malignant lung tissue microbiota. **a** Phylum-level taxonomic profiles. **b** Genus-level taxonomic profiles. **c** Kyoto Encyclopedia of Genes and Genomes (KEGG) module-level functional profiles. Each *vertical bar* represents a unique sample. Samples were ordered by anatomical sites shown below the figure. The *y-axis* shows the relative abundance of each phylum/genera/module. The average relative abundance (percentage) is shown in *parentheses* after each taxon or module. Only the most common taxa or modules are shown

The taxonomic and functional profiles of lung microbiota are presented in Fig. 1. Here, we defined the core member of lung microbiota if it is observed in 80 % of samples, regardless of the relative abundance. The core lung microbiota of non-malignant tissue samples at the phylum level included *Proteobacteria*, *Firmicutes*, *Bacteroidetes*, and *Actinobacteria* (Fig. 1a). At the genus level, the core lung tissue microbiota included five *Proteobacteria* genera: *Acinetobacter*, *Pseudomonas*, *Ralstonia*, and two unknown genus-level groups, one each from *Comamonadacea* and *Oxalobacteraceae* (Fig. 1b).

We also examined the NIAID (National Institute of Allergy and Infectious Diseases) class A–C pathogen genera and opportunistic “pathogens” as defined by the PATRIC database [15]. The potentially pathogenic genera *Staphylococcus*, *Streptococcus*, and *Burkholderia* were observed with low average relative abundances of around 2 %. Although *Pseudomonas*, which was frequently present in the lung tissue, is not included in the NIAID pathogen list, it could be pathogenic in immunosuppressed subjects. Other than these, “pathogens” were rarely observed in the non-malignant lung tissue (Additional file 1: Table S2).

Predicted functions based on Phylogenetic Investigation of Communities by Reconstruction of Unobserved States (PICRUSt) analysis of 16S rRNA gene taxonomic data are shown in Fig. 1c for the most abundant modules. The functional profiles exhibited less variation across individuals than found for taxonomic profiles (compare Fig. 1a and 1c).

### Analysis of negative controls

We sequenced four negative controls at the same time as our original lung tissue analysis to test PCR amplification and sequencing (“PCR negative controls”). Moreover, we sequenced an additional 20 negative controls that were subjected to DNA extraction and PCR amplification (“Extraction negative controls”). At the same time, we PCR-amplified and re-sequenced DNA from ten previously extracted lung tissue specimens. All samples were extracted by the same laboratory and the same laboratory technician, using the same kit and following the same extraction and PCR procedures we had used for the original lung tissue specimens. Finally, we sequenced a vagina sample and a fecal sample as positive controls. We found that:

1. The number of reads in all negative controls (44–351 reads) was much lower than the number of reads in lung tissue samples (1551–12,340 reads) and the positive samples (2703–58,201) (Additional file 1: Table S3).
2. We found five operational taxonomic units (OTUs) that were shared across all negative controls. None of these five OTUs were present in the lung tissue samples.
3. At the phylum level, there was a strong difference in microbial profiles between the lung tissue samples and the 20 extraction negative controls (*P* = 0.001, performed on the Euclidean distance of phylum-level profiles by non-parametric permutation multivariate analysis of variance (MANOVA), Adonis test with 1000 permutations). The lung tissue samples also differed from the four PCR negative controls, although the *P* value was not statistically significant (*P* = 0.1) because of the small sample size (*n* = 4) of these negative controls.
4. There were 173 OTUs shared between the negative controls and the lung tissue samples. When we excluded the shared 173 OTUs from the original analysis, all results regarding lung tissue remained virtually unchanged.

### Comparison of microbiotas from non-malignant lung tissue and other body sites

**Fig. 2:**
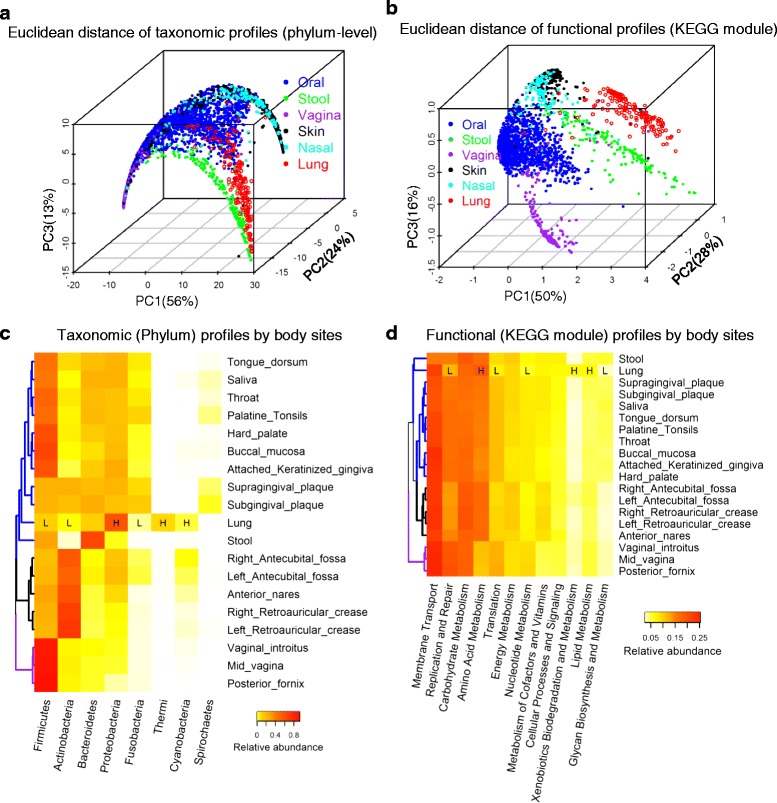
Comparison of microbiotas from non-malignant lung tissue and other human body sites (HMP 16S V3–V5 phase 1 data). **a**, **b** Principal coordinates analysis (PCoA) of Euclidean distance of phylum-level taxonomic profiles (**a**) and KEGG module functional profiles (**b**). The proportion of variance explained by each principal component is denoted in the corresponding *axis label*. **c**, **d** Phylum-level taxonomic profiles (**c**) and KEGG module functional profiles (**d**) by body site. The dendrogram shows similarity by body site based on the Euclidean distance of phylum-level/KEGG module profiles (average by body sites). Only phyla/modules with relative abundance >1 % are shown. *Branch colors* show different body sites (*blue*, oral, lung, and stool; black, nasal and skin; *purple*, vagina). “*H*” and “*L*” indicate that lung is significantly higher or lower, respectively, in relative abundance of the designated phylum/module compared with all the other body sites’ samples combined (*P* < 0.05 by Wilcoxon test with Bonferroni correction)

We compared the bacterial composition and abundance of non-malignant lung tissue with those of other body sites as established by the Human Microbiome Project (HMP) phase 1 using sequence data from the 16S rRNA gene regions V3–V5. We computed Euclidean distances between phylum/KEGG module relative abundance profiles, extracted the principal components of the corresponding distance matrix, and plotted the first three principal components to visualize samples (Fig. 2). The lung tissue microbiota formed a distinct cluster, largely separated from the oral microbiota and the microbiota commonly found at other body sites in healthy humans (Fig. 2a). The separation of the lung microbiota is even clearer based on functional profiles (Fig. 2b), for which almost no overlap was observed between lung and oral microbiota. Bray–Curtis distances provided similar results.

Figure 2c depicts the clustering of average phyla relative abundances by body site. The lung microbiota is distinct from that of other body sites in having a higher relative abundance of *Proteobacteria*, *Thermi*, and *Cyanobacteria*. Interestingly, *Thermi* had an average relative abundance of 8.8 % in lung but was rare in other body sites, with a highest average of 0.05 % in left antecubital fossa (Additional file 2: Figure S2). The Taq DNA polymerase used in this study was produced from *Escherichia coli*, not from *Thermus aquaticus*; therefore, it is unlikely that the *Thermi* observed in our samples were due to contamination. To confirm this, we re-sequenced five samples that originally included *Thermi* and five samples that originally had no *Thermi.* Five out of five positive samples remained positive in the re-sequencing data and five out of five negative samples remained negative in the re-sequencing data (Additional file 1: Table S4). These replication data argue strongly against laboratory contamination during PCR amplification or sequencing as the source of *Thermus* (*Thermi*) in the lung specimens, in which *Thermus* (*Thermi*) was one of the most abundant genera. In the repeated sequencing, the copy numbers of *Thermi* in the five positive cases were lower than in the original samples. This may be due to batch effects in the PCR amplification or because the DNA amount remaining for the replication assay was lower than the amount used for the original analysis.

Analogous clustering of predicted functions (Fig. 2d) shows that the lung microbiota had high relative abundance of the KEGG modules amino acid metabolism, xenobiotic biodegradation and metabolism, and lipid metabolism. Functional profiles and taxonomic profiles were correlated (Additional file 2: Figure S3).

### Demographic and clinical associations of non-malignant lung tissue microbiota

**Fig. 3:**
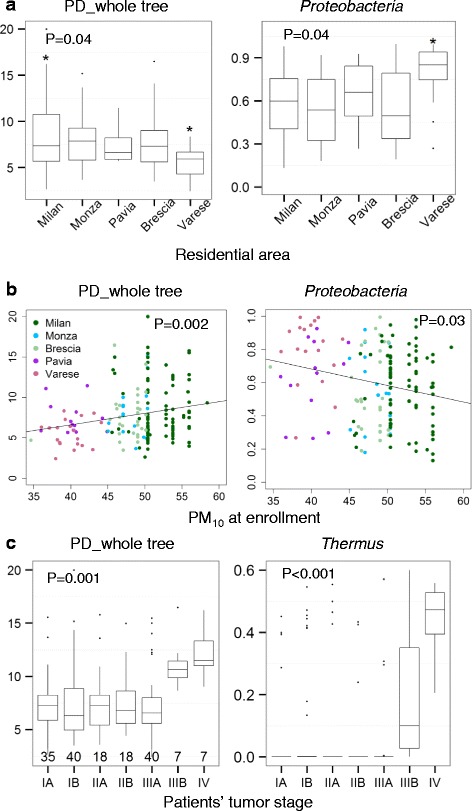
Non-malignant lung tissue microbiota in relation to participants’ residential areas (**a**), particulate matter 10 micrometers in diameter (PM_10_) at enrollment (**b**) and tumor stage (**c**). The *P* values shown in **a** and **c** are based on Kruskal–Wallis tests but were also validated in an adjusted linear regression model (model with residential area, history of chronic bronchitis, and tumor stage). The *P* values in **b** are based on a linear regression model with PM_10_, history of chronic bronchitis, and tumor stage in the model. The *asterisks* in **a** indicate areas significantly different from the overall mean. *Proteobacteria* for residential areas and air pollution and *Thermus* for tumor stage are the only taxa that showed significant association according to both an adjusted linear regression model and a Kruskal–Wallis test with Bonferroni correction

We found significant differences in taxonomic alpha diversity and *Proteobacteria* relative abundance by patient residence (Fig. 3a). In particular, samples from participants living in Varese, which has a low population density (1470 inhabitants/km^2^), had low alpha diversity and high *Proteobacteria* abundance, while samples from participants living in Milan, with a high population density (7389 inhabitants/km^2^), had high alpha diversity. Similar associations with alpha diversity and *Proteobacteria* relative abundance were found when plotted against atmospheric particulate matter 10 micrometers in diameter (PM_10_) concentrations at the time of participants’ enrollment (Fig. 3b), which is likely to reflect previous exposures [16]. These associations remained after regression adjustment for history of bronchitis and tumor stage (see below for associations with these factors). The regressions that included both PM_10_ concentrations and residential area indicated non-statistically significant main effects for both. No KEGG module/pathway relative abundance was associated with residential area or PM_10_ concentration (results not presented).

Analysis of beta diversity by residential area or PM_10_ MANOVA adjusted for history of bronchitis and tumor stage) showed a statistically significant association based on unweighted UniFrac distance (*P* = 0.009 and 0.006, respectively) but not on weighted UniFrac distance (*P* = 0.14 and 0.16, respectively). However, when both residential area and PM_10_ were included in the same model, PM_10_ (*P* = 0.003), but not residential area (*P* = 0.17), remained statistically significant.

We observed no statistically significant differences among microbiota from various anatomical locations in the lung (Additional file 1: Table S5). Samples from the principal bronchus had non-significantly higher taxonomic alpha diversity than samples from lung lobes (observed species (mean ± sd), 116.6 ± 53.6 in principal bronchus versus 84.0 ± 24.0 in lobes), but this test was based on only five principal bronchus samples. Alpha diversity was similar in different lobes of the lung (Additional file 1: Table S5).

We observed a significant positive association of pack-years of cigarette smoking with taxonomic alpha diversity (Shannon index, P_trend_ = 0.04 and observed species P_trend_ = 0.04). No other significant association for lung microbiota measures was observed by smoking status, years of smoking, or cigarettes per day (Additional file 1: Table S6).

Compared with patients with no history of previous lung diseases, patients with a history of emphysema, COPD, and pneumonia had similar levels of taxonomic alpha diversity (Additional file 1: Table S7), but patients with a history of bronchitis had significantly decreased alpha diversity (observed species, *P* = 0.05; PD_whole_tree, *P* = 0.02). No difference in beta diversity and relative abundance of any taxa was found between patients with and without previous lung diseases (data not shown). The dominant phylum, *Proteobacteria*, did not differ by previous disease status, including COPD (mean ± sd, 0.60 ± 0.22 and 0.62 ± 0.25 for patients with and without COPD, respectively). We also examined the association of spirometry-based lung function measures, including forced vital capacity, forced expiratory volume in 1 s, peak expiratory flow, and the ratio of forced vital capacity and forced expiratory volume in 1 s, with lung tissue microbial features, including alpha and beta diversity and taxa relative abundance, and found no association (data not shown).

Microbiota from non-malignant lung tissue had significantly increased PD_whole_tree, but not Shannon’s index in late stages IIIB and IV (Fig. 3c). Beta diversity (adjusted MANOVA analysis) was also significantly associated with tumor stage (*P* = 0.001 for both unweighted and weighted UniFrac). The genus *Thermus* (*Thermi*) had significantly higher abundance in stages IIIB and IV (Fig. 3c). With respect to predicted function, microbiota significantly differed by tumor stage in predicted KEGG modules and pathways (Additional file 2: Figure S4). Specifically, compared with patients in stages IA to IIIA, microbiota in stage IV patients had increased relative abundance for the excretory system module and the amino acid metabolism, aldosterone regulated sodium reabsorption, and amoebiasis pathways. Moreover, microbiota in patients with both stage IIIB and IV had reduced abundance for signal transduction (Additional file 2: Figure S4).

We observed no difference in taxonomic alpha diversity or beta diversity by metastasis status after diagnosis. However, the non-malignant samples from patients who developed metastases, compared with those without metastases, had significantly increased relative abundance of *Legionella* (*Proteobacteria*) (mean ± sd, 0.003 ± 0.008 versus 0.001 ± 0.004, *P*(Bonferroni) = 0.01).

### Difference between lung tumor and non-malignant tissue microbiotas

**Fig. 4:**
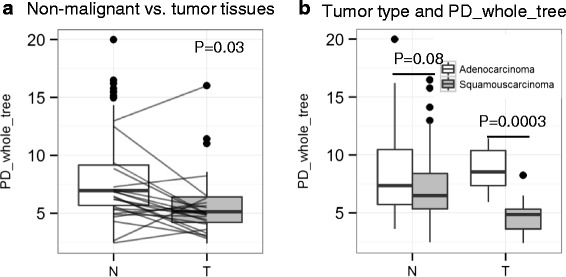
Comparison of non-malignant (*N*) and tumor (*T*) tissue microbiotas. **a** Non-malignant and tumor tissue microbiotas significantly differ in taxonomic alpha diversity. The *P* value was computed by the signed rank Wilcoxon test based on paired samples and was also confirmed by the bootstrap analysis (see “Statistical methods”) of all paired and unpaired samples (*P* < 0.001). **b** Comparison of microbiota by tumor morphology in non-malignant and tumor tissues. Taxonomic alpha diversity (PD_whole_tree) is statistically significantly higher in patients with adenocarcinoma in microbiota from the tumor samples but not from non-malignant samples. *P* values are based on Wilcoxon tests

We had fresh frozen tumor tissue samples from 56 subjects. After excluding samples with <1000 reads per sample, we included data from 31 tumor samples for comparison with non-malignant tissues. Several measures of alpha diversity were significantly higher in non-malignant than in tumor lung tissues (e.g., PD_whole_tree; Fig. 4a). In addition, microbiota differed significantly between non-malignant and tumor tissue by tumor histology (Fig. 4b). While no major differences were observed in non-malignant tissue by tumor histology, the tumor tissues with adenocarcinoma had significantly higher phylogenetic diversity (PD_whole_tree; Fig. 4b), increased relative abundance of *Thermus* (*Thermi*; 0.285 ± 0.231 versus 0.017 ± 0.084, *P*(Bonferroni) = 0.02), and decreased relative abundance of *Ralstonia* (*Proteobacteria*; 0 ± 0.001 versus 0.026 ± 0.046, *P*(Bonferroni) = 0.04) than tumor tissues with squamous cell carcinoma. In contrast, weighted UniFrac distance did not significantly differ between non-malignant and tumor tissue and was lower within than between subjects (Additional file 2: Figure S5).

## Discussion

In the largest study of human non-malignant lung tissue to date, we describe the taxonomic and functional profiles of lung tissue microbiota. The lung tissue microbiota was clearly distinct from the microbiotas reported at other body sites (oral cavity, nasal cavity, gut, skin, and vagina). Moreover, it showed increased alpha diversity with environmental exposures such as air particulates, residence in high population density areas, and pack-years of tobacco smoking. Microbiota also varied by clinical endpoints, with increased alpha diversity in non-malignant lung tissue from advanced stages of cancer and decreased alpha diversity in lung affected by chronic bronchitis. Microbiota alpha diversity also significantly differed between non-malignant and tumor lung tissue.

Most previous studies on the airway microbiota were based on BAL, bronchoscopic brushing, or sputum samples [7, 10, 17–26]. A common concern of these samples is that they may be contaminated by the upper respiratory tract and oral microbiota [8]. In our study, samples were surgically resected from lung tissue distant from the tumors and with no evidence of tumor nuclei. We found that five *Proteobacteria* genera had high relative abundance in lung tissue with and without COPD or other lung diseases and each genus was found in 80 % of samples. In contrast, previous studies of BAL showed high abundance of genera *Prevotella* (*Bacteroidetes*), *Streptococcus* (*Firmicutes*) and *Veillonella* (*Firmicutes*) (Additional file 1: Table S8), which are commonly found in the oral cavity (Additional file 1: Table S9). Reassuringly, the only four previous, very small studies of lung tissue also indicated members of the phylum *Proteobacteria* as predominant (Additional file 1: Table S6) [10–13]. Notably, we found that *Thermi*, although present in only 27 % of subjects, had high relative abundance in samples from these subjects (mean ± sd, 32 ± 20 %). To our knowledge, only one BAL-based study reported the presence of high relative abundance of *Thermi (Thermus,* ~96 %*)* in one healthy subject [24]. Moreover, our data clearly show that the lung microbiota is distinct from digestive tract microbiota of healthy subjects, even at high taxonomic (phylum) and functional (KEGG module) level (Fig. 2a, b). Taken together these data suggest that the lung microbiota is unique.

A concern is that our lung tissue assays might be contaminated during DNA extraction, PCR amplification, or sequencing. Analyses of 24 negative controls and re-amplification and re-sequencing of DNA from ten previously analyzed lung specimens argue strongly against distortion of our results by contamination. Although *Thermi* has high resistance to environmental hazards [27] (*Thermus acquaticus* can survive at 50 to 80 °C and is the source of thermostable Taq DNA polymerase, which is commonly used for DNA amplification (https://en.wikipedia.org/wiki/Thermus_aquaticus)), we found no evidence for *Thermi* contamination. Repeat amplification and sequencing of five initially *Thermi-*negative DNA samples yielded five negative results and five initially *Thermi-*positive DNA samples yielded five repeat positive results. More generally, when all the 173 OTUs that were common to 24 negative control samples and to any lung cancer sample were excluded, the lung cancer analyses were virtually unchanged.

We found a positive association between microbiota alpha diversity in the lung and subjects’ residential area (from low to high density population), with evidence that this association reflected exposure to air pollution (PM_10_). We did not find significant differences in the lung tissue microbiota by smoking status, smoking intensity, or lifetime smoking duration, probably because there was no great variability across study subjects since most of them were heavy smokers. However, we did find significantly higher alpha diversity with increased pack-years of cigarette smoking. Together, these findings suggest that the lung microbiota may be altered by cumulative exposure to tobacco smoke and other air pollutants or life style conditions linked to high density population. Previously, analysis of 16 sputum samples revealed higher alpha diversity in samples from women in China who used smoky coal for cooking and heating compared with those using smokeless coal. Also, increased diversity and altered abundances of certain taxa in smokers’ versus nonsmokers’ subgingival samples have been shown [28]. Chronic inhalation of dust or tobacco-related particles could allow increased diversity by impeding the dispersion and clearance of microbes from the bronchopulmonary system. Alternatively, particulates in the air could function as vectors for inhalation of microbes, as suggested by a study in which the dust from households with a dog or a cat had higher microbial diversity compared with the dust from households with no furred pets [29].

Long lasting and repetitive irritation of inhaled substances such as tobacco smoke, dust and silica may promote the development of bronchitis. Early clinical features of bronchitis include hyper-secretion of mucus and hypertrophy of sub-mucosal glands, eventually leading to chronic airway obstruction and possibly secondary growth of specific bacteria. This could be related to the lower alpha diversity we identified in subjects affected by chronic bronchitis. Clearly, larger clinical studies and investigations in model systems are warranted to further understand the viability of the microbiota, its role in chronic diseases, and its potential use for prevention and treatment strategies.

Despite painstaking exclusion of samples adjacent to tumors, we observed alteration of the lung microbiota in non-malignant tissue samples from subjects with advanced tumor stages (IIIB and IV). Specifically, these samples had higher phylogenetic diversity, high relative abundance of *Thermus* (*Thermi*), and increased/decreased abundance of several functional modules compared with samples from patients with earlier stages of lung cancer. Moreover, subjects who developed metastases had high relative abundance of *Legionella* (*Proteobacteria*)*.* These data suggest that *Thermus* and *Legionella* might play a role in tumor progression, partially through the different microbiota functions, e.g., reduced signal transduction, increased excretory systems, amino acid metabolism, aldosterone-regulated sodium reabsorption, or amoebiasis pathways. Alternatively, tumor progression could affect the microenvironment and microbiota of a larger surrounding area. Given our collection methods and careful histological review, it is unlikely that the non-malignant tissue samples from advanced-stage subjects were contaminated by their tumor microbiota. In fact, the tumor microbiota showed low phylogenetic diversity that was unlike the corresponding non-malignant tissue. Moreover, while the microbiota differed in the tumor samples between subjects with adenocarcinoma and squamous cell carcinoma, the corresponding microbiota in these subjects’ non-malignant samples did not differ. This suggests that the microbiota from non-malignant lung tissue samples are different from that of the tumor lung tissue samples, as has been previously shown for tumor/non-malignant samples from colorectal cancer patients [30]. It will be important to explore whether the microbiota in non-malignant lung tissue from advanced disease stages, or in tumor tissue, plays a role in tumor progression or is just a passive byproduct of tumor progression.

This study includes noteworthy strengths and limitations. It is the largest study of the non-malignant lung tissue microbiota to date. Moreover, we used uniform surgical procedures performed under sterile conditions for obtaining the surgical samples, which were frozen immediately. Also, we examined a comprehensive list of detailed and validated epidemiological and clinical variables in relation to features of the lung microbiota. Furthermore, we performed rigorous analysis to take into account correlations across subjects and paired samples and sequenced negative controls to exclude the possibility of contamination. One important caveat is that we compared our lung data with that of healthy individuals enrolled in the HMP. The two populations are very different, in age and health status, with young and extremely healthy individuals in the HMP study and older (average age 67 years) lung cancer patients in this study. Further, DNA extraction techniques, 16S rRNA gene primers, and sequencing platforms were all different and have been previously shown to potentially introduce some biases [31]. However, we minimized the effects of these differences by restricting our comparisons to the highest and least variable taxonomic level (phylum level) and functional entity (KEGG module). In addition, a meta-analysis of microbiota studies revealed that differences in microbial populations across body sites are significantly larger than those driven by the experimental protocols, age, geography, and other population characteristics [32]. Another limitation is that we could not study the effect of antibiotic use on the lung microbiota because most patients were treated with antibiotics at the time of surgery. In addition, as in most microbiota studies, we do not know whether the DNA we studied corresponded to living or dead bacteria and further studies are needed to address this issue. Furthermore, because we used a stringent threshold (1000 reads) to obtain reliable estimates of microbiota relative abundance, we had to exclude ~23 and ~45 % of samples from non-malignant and tumor sites, respectively. If we had used a less stringent threshold, e.g., 500 reads (which is still larger than the number of reads identified in most negative controls), we would have excluded only 13 and 25 % of samples, respectively. We opted to use a more stringent threshold since this is the first large study of microbiota in human lung tissue and it is important to report data with enough reads to characterize the lung bacterial community accurately. Finally, although we found no association between microbiota features and COPD or spirometry-based lung function, we cannot exclude that the lung cancer patients had abnormalities in their lungs that could affect our results. Our findings in lung cancer patients, although based on non-malignant tissues, may not be completely applicable to healthy subjects.

## Conclusions

In the largest study of non-malignant lung tissue to date, we show that the lung microbiota has distinct features that differ from those of the oral cavity and other body sites and is dominated by *Proteobacteria* (60 %). The lung microbiota is affected by exposure to air pollution and tobacco smoking and is different in subjects with chronic bronchitis or advanced tumors. The genus *Thermus* is more abundant in tissue from advanced stage patients, while *Legionella* is higher in patients who develop metastases. Further studies in lung tissue and animal model systems are necessary to investigate the role of microbiota in the development of lung diseases and whether it can be exploited for treatment purposes.

## Methods

### Subject characteristics and epidemiological and clinical data collection

The study is nested in the Environment and Genetics in Lung cancer Etiology study (EAGLE), which was described in detail previously [33]. In brief, EAGLE is an integrated population-based study of lung cancer with the aim to capture the major risk factors and genetic basis of lung cancer. The study was conducted in the Lombardy region of Italy and the catchment area included five cities (Milan, Monza, Brescia, Pavia, and Varese) and their surrounding municipalities (see Additional file 2: Figure S1 for a map). The lung cancer cases were enrolled from 13 hospitals that covered approximately 80 % of lung cancer cases from the catchment area. The epidemiological data were collected by a computer assisted personal interview and a self-administered questionnaire at the time of lung cancer diagnosis. The clinical data were collected by physicians from the clinical charts and hospital discharge records. The average annual atmospheric concentration (μg/m^3^) of particulate matter of 10 micrometers in diameter (PM_10_) in the subjects’ cities of residence during the year of their enrollment was estimated by combining land-use regression data with aerosol optical depth data from the MODIS (Moderate Resolution Imaging Spectroradiometer) instrument onboard the National Aeronautics and Space Administration’s Terra satellite [34].

### Biospecimen collection

Lung tissue samples were snap-frozen in liquid nitrogen within 20 minutes of surgical resection. Surgeons and pathologists were together in the surgery room at the time of resection and sample collection to ensure correct sampling of tissue from the tumor, the area adjacent to the tumor, and an additional area distant from the tumor (~1–5 cm), without adversely affecting the participant. The precise site of tissue sampling was indicated on a lung drawing and the pathologists classified the samples as tumor, adjacent lung tissue, and distant non-involved lung tissue. For the current study, we used lung tissue sampled from an area distant from the tumor (defined here as “non-malignant lung tissue”) to reduce the potential for local cancer field effects. For each subject, usually more than one non-malignant lung tissue sample was collected and at least one sample was examined by a pathologist to confirm the absence of tumor nuclei. All the tools and materials in contact with the lung tissues were sterile. Based on sample availability, we selected 233 non-malignant and 56 tumor samples. Results are based on 165 non-malignant and 31 tumor samples after quality control-determined exclusions.

### 16S rRNA gene sequence analysis

Fresh frozen lung tissue samples remained frozen while approximately 30 mg was subsampled for DNA extraction into pre-chilled 2.0 ml microcentrifuge tubes. Lysates for DNA extraction were generated by incubating 30 mg of tissue in 1 ml of 0.2 mg/ml Proteinase K (Ambion) in DNA lysis buffer (10 mM Tris-Cl (pH 8.0), 0.1 M EDTA (pH 8.0), and 0.5 % (w/v) SDS) for 24 h at 56 °C with shaking at 850 rpm in Thermomixer R (Eppendorf). DNA was extracted from the generated lysate using the QIAamp DNA Blood Maxi Kit (Qiagen) according to the manufacturer’s recommendation. The V3–V4 regions of the 16S rRNA gene were amplified and sequenced on an Illumina MiSeq instrument using the 300 paired-end protocol at the Institute of Genome Sciences, Genomic Resource Center, University of Maryland School of Medicine as described previously [35]. We included two positive controls (one fecal and one vaginal sample) to examine the performance of the sequence run, 20 negative controls to examine the potential contamination by DNA extraction and PCR reagents, and four negative controls to exclude contamination during the PCR amplification process. We showed that our results are not affected by potential contamination.

Sequence reads were processed to remove low quality reads, specifically reads with average quality less than 20 over a 30-bp window based on the Phred algorithm. These were trimmed before the first base of the window and re-evaluated for length. Also removed were paired reads that had at least one of the reads with length less than 75 % of its original length, reads with less than 60 % similarity to Greengenes reference version 13_8 [36], and chimera reads (identified using UCHIME [37]). The remaining reads were clustered into OTUs at 97 % identity using the command pick_open_reference_otus.py in the software package Quantitative Insights into Microbial Ecology (QIIME 1.8.0) [38]. The default parameters were used except method of usearch61 and percent_subsample of 0.1. OTUs with only one read or in only one sample were excluded.

Taxonomic alpha diversity was estimated as the number of 97 % identical OTUs (Observed_species), Shannon’s Index (using information of the relative abundance of observed species) [39] and phylogenetic diversity whole tree (PD_whole_tree, using information on both the relative abundance and phylogenetic tree of observed species) [40] by averaging over 20 rarefied tables (1000 reads/sample). Taxonomic beta diversity was measured as unweighted (presence/absence of observed species) and weighted UniFrac distance (also using information on the relative abundance of observed species) based on the OTU table [41]. Relative abundance of taxa was calculated from unrarefied OTU table.

We downloaded the V3–V5 16S rRNA gene sequence data from the Human Microbiome Project (HMP, phase 1; http://hmpdacc.org/) and processed for comparison [14]. The HMP data include 138–1623 samples with at least 1000 sequence reads per sample from each studied site (including oral cavity, nasal cavity, skin, stool, and vagina). Euclidean distance and Bray–Curtis distance were calculated based on phylum-level relative abundance for comparison between lung tissue and other body sites.

### Functional prediction from 16S rRNA gene sequence data

PICRUSt 1.0.0 was used to predict the function of the microbiota from the 16S rRNA gene sequence taxonomic data for both the HMP dataset and this study using the KEGG database as reference [42, 43]. PICRUSt requires the use of Greengenes reference version 13_5 [36]. Therefore, we reprocessed the sequence data in QIIME as previously but using Greengenes reference version gg_13_5 [36].

Euclidean distance and Bray–Curtis distance were calculated using the rarified KEGG orthologs (KO) table (430,000 predicted reads per sample) for comparison among lung samples and the KEGG module table for comparison with other body sites. Relative abundance of modules/pathways was calculated from unrarefied KO table.

### Statistical methods

All statistical analyses were performed in the R software (R Foundation for Statistical Computing, Vienna, Austria; http://www.R-project.org/). In boxplots, the black central lines represent the median and box edges the first and third quartiles. Wilcoxon rank-sum and Kruskal–Wallis tests were used for differences between categories, and Spearman correlation test was used for association of continuous variables. For the variables that showed significant associations with microbiome features, we used multiple linear regression models with microbiota measurements as the dependent variable to test the association while adjusting for other covariates (residential area, history of bronchitis, and tumor stage). For comparisons between non-malignant and tumor samples, the Wilcoxon signed-rank test was used for paired samples. For comparisons that included both paired and unpaired samples, individuals were stratified into three categories, those with tumor tissue only, those with non-malignant tissue only, and those with both types of tissue. A bootstrap was performed by resampling individuals with replacement within these strata to estimate the variances of mean differences. Jackknife analyses likewise removed individuals one at a time from these strata to account for correlations among means. Only the taxa (phylum, class, order, family, or genus) with relative abundance greater than 0.001 in at least 10 % of the samples were included in the analysis. Unless otherwise indicated, *P* values were Bonferroni-adjusted for multiple comparisons (R command P.adjust).

MANOVA in R (Adonis method in R Package Vegan [44]) was used to examine the association between beta diversity and individual epidemiological and clinical variables, adjusting for the variables showing significant associations with microbiota (including subjects’ residential area, history of bronchitis, and tumor stage). For some comparisons of beta-diversity, the jackknife procedure was used to compute variances that allowed for correlations within and between subjects.

## Abbreviations

BAL, bronchoalveolar lavage; COPD, chronic obstructive pulmonary disease; EAGLE, Environment and Genetics in Lung cancer Etiology study; HMP, Human Microbiome Project; KO, KEGG orthologs; MANOVA, Non-parametric permutation multivariate analysis of variance; NIAID, National Institute of Allergy and Infectious Diseases; OTU, operational taxonomic unit; PICRUSt, Phylogenetic Investigation of Communities by Reconstruction of Unobserved States; PM, particulate matter; sd, standard deviation

## Additional files

Additional file 1: Supplementary Tables S1 through S9. (DOC 303 kb) Additional file 2: Supplementary Figures S1 through S5. (PDF 630 kb)
