# Supplementary material for: Characterizing human lung tissue microbiota and its relationship to epidemiological and clinical features
Source: Genome Biol. 2016 Jul 28;17:163. doi: 10.1186/s13059-016-1021-1 (PMC4964003; doi:10.1186/s13059-016-1021-1)
Supplement: Additional file 1: — Supplementary Tables S1 through S9. (DOC 303 kb) [file 13059_2016_1021_MOESM1_ESM.doc]

**Additional file 1**

**Table S1**. Characteristics of the study subjects by tissue type

|  |  | **Non-malignant (n=165)*** | **Tumor (n=31)*** |
| --- | --- | --- | --- |
| **Sex** | Female | 28(17%) | 5(16%) |
| **Age** |  | 66.6(62.1 - 72.6) | 66.4(63.8 - 71.3) |
| **Residential area** | Brescia | 29(18%) | 0 |
|  | Milan | 88(53%) | 20(65%) |
|  | Monza | 16(10%) | 2(6%) |
|  | Pavia | 13(8%) | 2(6%) |
|  | Varese | 19(12%) | 7(23%) |
| **Air pollution (PM10)** |  | 50.2(46.1 - 52.9) | 50.0(42.5 - 50.4) |
| **Smoking status** | Never smoker | 9(5%) | 2(6%) |
|  | Former smoker | 71(43%) | 10(32%) |
|  | Current smoker | 84(51%) | 17(55%) |
| **Cigarettes per day** |  | 21.9(15.0 - 29.1) | 24.2(13.1 - 31.0) |
| **Smoking total duration (years)** |  | 43.5(36.0 - 53.0) | 44.2(37.0 - 52.5) |
| **Pack years** |  | 45.3(27.8 - 60.0) | 52.7(24.0 - 81.0) |
| **History of COPD**  **(by spirometry)** | Yes | 75(45%) | 12(39%) |
| **History of Bronchitis** | Yes | 28(17%) | 5(16%) |
| **History of Emphysema** | Yes | 16(10%) | 2 (6%) |
| **History of Pneumonia** | Yes | 25(15%) | 7(23%) |
| **Anatomical site** | Principal bronchus | 5(3%) | 1(3%) |
|  | Upper lobe | 95(58%) | 14(45%) |
|  | Middle lobe | 9(5%) | 1(3%) |
|  | Lower lobe | 55(33%) | 14(45%) |
| **Tumor morphology** | Squamous carcinoma | 63(38%) | 25(81%) |
|  | Adenocarcinoma | 97(59%) | 6(19%) |
|  | Mixed types | 5(3%) | 0 |
| **Cancer stage** | IA | 35(21%) | 8(26%) |
|  | IB | 40(24%) | 7(23%) |
|  | IIA | 18(11%) | 2(6%) |
|  | IIB | 18(11%) | 4(13%) |
|  | IIIA | 40(24%) | 10(32%) |
|  | IIIB | 7(4%) | 0 |
|  | IV | 7(4%) | 0 |
| **Metastasis** | Yes | 83(50%) |  |
| **Survival (weeks)** |  | 201.9(94.6 - 307.8) | 196.3(84.2 - 319.9) |

* The number (percentage) or mean (interquartile) value is listed for each characteristic.

**Table S2.** Relative abundances of NIAID categorized 'Pathogen' genus in non-malignant lung tissues

|  | Median (interquartile) | Mean |
| --- | --- | --- |
| *Staphylococcus* | 0.002(0-0.019) | 0.021 |
| *Streptococcus* | 0.002(0-0.018) | 0.020 |
| *Burkholderia* | 0.008(0.001-0.022) | 0.016 |
| *Bacillus* | 0(0-0) | 0.004 |
| *Helicobacter* | 0(0-0) | 0.001 |
| *Clostridium* | 0(0-0) | 0.001 |
| *Campylobacter* | 0(0-0) | 0.000 |
| *Mycobacterium* | 0(0-0) | 0.000 |
| *Coxiella* | 0(0-0) | 0.000 |
| all | 0.04(0.02-0.08) | 0.06 |

**Table S3.** Reads analysis in lung samples and negative control samples. Median (interquartile range) of reads in each group (for the 4 PCR negative controls we list the reads for each sample)

| Original analysis | | |
| --- | --- | --- |
|  | Lung (n=196; including 165 non tumor tissue and 31 tumor tissue) | PCR negative controls (n=4) |
| Total number of reads per sample | 2898 (1734-4654) | 44|159|41|164 |
| Additional controls | | |
|  | Lung replicates (n=10) | Extraction negative controls (n=20) |
| Total number of reads per sample | 1. (1551-12340) | 20 (163-351) |

Table S4: Number of *Thermi* reads in 10 original and replicated lung tissue samples

| Sample ID | First analysis | Second analysis |
| --- | --- | --- |
| MSB000097 | 0 | 0 |
| MSB000105 | 0 | 0 |
| MSB000113 | 0 | 0 |
| MSB000121 | 0 | 0 |
| MSB000129 | 0 | 0 |
| MSB000242 | 2243 | 124 |
| MSB000236 | 2531 | 561 |
| MSB000237 | 1409 | 308 |
| MSB000239 | 1364 | 2 |
| MSB000263 | 1914 | 511 |

**Table S5.** Association between anatomical site and microbiota measures in non-malignant lung tissues

|  | **Principal bronchus** | **Upper lobe** | **Middle lobe** | **lower lobe** | **P** | **P(Bonferroni)** |
| --- | --- | --- | --- | --- | --- | --- |
| Observed_species | 116.6±53.6 | 85.3±25.0 | 85.5±20.4 | 81.6±23.0 | 0.43 |  |
| Shannon | 5.1±1.0 | 4.5±0.9 | 4.5±1.0 | 4.3±0.8 | 0.36 |  |
| PD_whole_tree | 9.6±5.0 | 8.0±3.2 | 8.4±3.9 | 7.4±3.1 | 0.58 |  |
| *f__Bradyrhizobiaceae.Other* | 0.003±0.006 | 0.004±0.02 | 0.005±0.006 | 0.005±0.013 | 0.00 | 0.15 |
| *f_Bradyrhizobiaceae* | 0.003±0.006 | 0.005±0.021 | 0.008±0.011 | 0.005±0.013 | 0.01 | 0.33 |
| *f__Rhodocyclaceae* | 0.026±0.023 | 0.016±0.045 | 0.015±0.016 | 0.006±0.013 | 0.03 | 1 |
| *g__Sphingobium* | 0.002±0.004 | 0.006±0.016 | 0.03±0.038 | 0.01±0.038 | 0.04 | 1 |
| *f__Sphingomonadaceae.Other* | 0±0.001 | 0.002±0.006 | 0.003±0.004 | 0.002±0.005 | 0.05 | 1 |

Note: The mean ± standard deviation were listed for each category; P value is based on the Kruskal-Wallis test and P(Bonferroni) is the Bonferroni-corrected P value. All the taxa belong to *Proteobacteria*.

**Table S6**. Association between smoking variables and taxonomic alpha diversity of non-malignant lung tissue microbiota

| **Smoking status** | | | | | |
| --- | --- | --- | --- | --- | --- |
|  | **never smokers (n=9)** | **former smoker (n=71)** | **current smoker (n=84)** | **P_trend** | |
| Observed_species | 83.3±22.4 | 84.1±27.1 | 85.8±25.2 | 0.66 | |
| Shannon | 4.3±0.7 | 4.5±0.9 | 4.5±0.9 | 0.89 | |
| PD_whole_tree | 8.1±3.3 | 7.8±3.5 | 7.9±3.1 | 0.98 | |
| **Smoking Status (redefined)** | | | | | |
|  | **never and smokers quit for >=10 years (n=49)** | **former smokers quit for <10 years (n=31)** | **current smokers (n=84)** | **P_trend** | |
| Observed_species | 81.1±24.2 | 88.7±29.5 | 85.8±25.2 | 0.37 | |
| Shannon | 4.5±0.8 | 4.6±1.0 | 4.5±0.9 | 0.95 | |
| PD_whole_tree | 7.5±3.2 | 8.3±3.8 | 7.9±3.1 | 0.60 | |
| **Number of cigarette per day** | | | | | |
|  | **<20 (n=59)** | **20 - 30 (n=57)** | **>=30 (n=37)** | **P_trend** | |
| Observed_species | 85.5±23.4 | 82.6±23.6 | 88±33.2 | 0.75 | |
| Shannon | 4.5±0.8 | 4.4±0.9 | 4.6±0.9 | 0.74 | |
| PD_whole_tree | 8.1±3.4 | 7.5±3.0 | 8.0±3.5 | 0.72 | |
| **Smoking total duration in years** | | | | | |
|  | **<40 years (n=53)** | **40 - 51 years (n=53)** | **>=51 years (n=46)** | **P_trend** | |
| Observed_species | 84.7±29.9 | 84.1±23.9 | 86.2±24.6 | 0.78 | |
| Shannon | 4.4±0.9 | 4.5±0.8 | 4.5±0.9 | 0.59 | |
| PD_whole_tree | 7.9±4.0 | 7.7±2.8 | 8.0±2.9 | 0.82 | |
| **Pack years** | | | | | |
|  | **<36 (n=52)** | **36 - 55 (n=50)** | **>=55 (n=50)** | **P_trend** | **P_trend*** |
| Observed_species | 81.3±22.4 | 83.8±24.5 | 91.2±29.8 | 0.06 | **0.04*** |
| Shannon | 4.3±0.9 | 4.5±0.8 | 4.7±0.7 | **0.03** | **0.04*** |
| PD_whole_tree | 7.7±3.4 | 7.6±3.1 | 8.3±3.3 | 0.39 | 0.22* |

Note: The smoking variables were categorized as shown and the number in the brackets are sample size for each category. The mean ± standard deviation were listed for each category; *P value based on multivariate model adjusted for age, sex and history of bronchitis.

**Table S7.** Association between taxonomic alpha diversity in non-malignant lung tissue and history of lung diseases

|  | **Without the history*** | **With the history*** | **P (Wilcoxon)** | **Multivariate^†^** | |
| --- | --- | --- | --- | --- | --- |
|  |  |  |  | **Coefficient** | **P value** |
| **Bronchitis (n=28)** | | | | | |
| Observed_species | 86.6 ± 27.2 | 75.4 ± 19.5 | **0.02** | -11.4 | **0.05** |
| Shannon | 4.5 ± 0.9 | 4.3 ± 0.8 | 0.25 | -0.2 | 0.38 |
| PD_whole_tree | 8.0 ± 3.3 | 6.9 ± 2.9 | **0.03** | -1.6 | **0.02** |
| **Emphysema (n=16)** | | | | | |
| Observed_species | 85.4 ± 26.2 | 82.6 ± 25.5 | 0.54 |  |  |
| Shannon | 4.5 ± 0.9 | 4.5 ± 0.7 | 0.9 |  |  |
| PD_whole_tree | 7.9 ± 3.3 | 7.3 ± 3.1 | 0.4 |  |  |
| **COPD (n=75)** | | | | | |
| Observed_species | 83.9 ± 25.5 | 84.1 ± 23.9 | 0.80 |  |  |
| Shannon | 4.4 ± 0.9 | 4.5 ± 0.8 | 0.29 |  |  |
| PD_whole_tree | 7.8 ± 3.3 | 7.5 ± 3.0 | 0.61 |  |  |
| **Pneumonia (n=25)** | | | | | |
| Observed_species | 84.9 ± 26.2 | 81.7 ± 22.2 | 0.72 |  |  |
| Shannon | 4.5 ± 0.9 | 4.4 ± 0.7 | 0.41 |  |  |
| PD_whole_tree | 7.8 ± 3.3 | 7.6 ± 3.2 | 0.58 |  |  |

* Mean ± standard deviation are listed for each category.

† Multivariate model adjusted for participants’ residential area and cancer stage.

**Table S8.** Microbiota components in BALs of healthy subjects or lung tissue samples

| Tissue type | Sample | Phylum | Geneus |
| --- | --- | --- | --- |
| Lung tissue (this study) | 165 non-tumor lung tissue from lung cancer patients | *Proteobacteria (59.7%)* | *Proteobacteria.Acinebobacter (11.9%)* |
|  |  | *Firmicutes(10.6%)* | *Proteobacteria.Comamonadaceae(11.6%)* |
|  |  | *Bacteroidetes(10.4%)* | *Thermi.Thermus(8.7%)* |
|  |  | *Thermi(8.8%)* | *Proteobacteria.Pseudomonas(7.1%)* |
|  |  | *Actinobacteria(3.9%)* | *Proteobacteria.Oxalobacteraceae(5.5%)* |
|  |  | *Cyanobacteria (1.3%)* | *Proteobacteria.Ralsonia(2.1%)* |
|  |  |  | *Firmicutes.Staphylococcus(2.1%)* |
|  |  |  | *Firmicutes.Streptococcus(2.0)* |
| BAL | 28 healthy subjects | *Bacteroidetes (38%)* | *Bacteroidetes.Prevetella (28%)* |
|  |  | *Firmicutes (21%)* | *Firmicutes.Streptococcus (16%)* |
|  |  | *Proteobacteria (18%)* | *Proteobacteria.Neisseria (7.8%)* |
|  |  | *Fusobacteria (6.2%)* | *Fusobacteria.Fusobacterium (5.3%)* |
|  |  | *Actinobacteria (2.6%)* | *Firmicutes.Veillonella (3.3%)* |
|  |  |  | *Bacteroidetes.Porphyromonas (2.3%)* |
|  |  |  | *Firmicutes.Lactobacillus (1.6%)* |
|  |  |  | *Proteobacteria.Halomonas (1.5%)* |
|  |  |  | *Bacteroidetes.Capnocytophaga (1.3%)* |
|  |  |  | *Actinobacteria.Actinomyces(1%)* |
| BAL | 26 non-transplant control without lung diseases | *Bacteroidetes* | *Bacteroidetes.Prevotellaceae* |
|  |  | *Firmicutes* | *Firmicutes.Veillonellaceae* |
|  |  | *Proteobacteria* | *Firmicutes.Streptococcaceae* |
|  |  | *Fusobacteria* | *Firmicutes.Lachnospiraceae* |
|  |  |  | *Proteobacteria.Neisseriaceae* |
|  |  |  | *Bacteroidetes.Porphyromonadaceae* |
|  |  |  | *Proteobacteria.Pseudomonadaceae* |
|  |  |  | *Proteobacteria.Enterobacteriaceae* |
|  |  |  | *Firmicutes.Staphylococcaceae* |
|  |  |  | *Proteobacteria.Alcaligenaceae* |
| Bronchoscopic brushing of LUL | 8 Controls | *Bacteroidetes* | *Bacteroidetes.Prevotella* |
|  |  | *Firmicutes* | *Firmicutes.Streptococcus* |
|  |  | *Proteobacteria* | *Firmicutes.Veilonella* |
|  |  | *Fusobacteria* | *Proteobacteria.Haemophilus* |
|  |  | *Actinobacteria* | *Proteobacteria.Neisseria* |
|  |  |  | *Actinobacteria.Corynebacterium* |
|  |  |  | *Firmicutes.Staphylococcus* |
| BAL | 6 healthy subjects | *Firmicutes* | *Bacteroidetes.Prevetellaceae* |
|  |  | *Bacteroidetes* | *Firmicutes.Veilonellaceae* |
|  |  | *Proteobacteria* | *Firmicutes.Streptococcaceae* |
|  |  | *Actinobacteria* | *Proteobacteria.Neisseriaceae* |
|  |  | *Fusobacteria* | *Fusobacteria.Fusobacteriaceae* |
|  |  |  | *Actinobacteria.Corynebacterium* |
|  |  |  | *Firmicutes.Lachnospiraceae* |
| BAL | 3 never smokers, 7 healthy smoker, 4 COPD | *Proteobacteria* | *Proteobacteria.Pseudomonas* |
|  |  | *Bacteroidetes* | *Firmicutes.Streptococcus* |
|  |  | *Firmicutes* | *Bacteroidetes.Prevotella* |
|  |  | *Fusobacteria* | *Fusobacteria.Fusobacterium* |
|  |  | *Actinobacteria* | *Proteobacteria.Haemophilus* |
|  |  |  | *Firmicutes.Veillonella* |
|  |  |  | *Bacteroidetes.Porphyromonas* |
| BAL | 10 healthy control | *Actinobacteria* |  |
|  |  | *Firmicutes* |  |
|  |  | *Proteobacteria* |  |
|  |  | *Termi (Thermus)* |  |
|  |  | *Bacteroidetes* |  |
| BAL | 9 never smokers, 14 former smokers and 6 current smokers, no lung disease |  | *Firmicutes.Veilonella* |
|  |  |  | *Bacteroidetes.Prevetella* |
| BAL | 64 Healthy subjects |  | *Bacteroidetes.Prevotella* |
|  |  |  | *Firmicutes.Streptococcus* |
|  |  |  | *Firmicutes.Veillonella* |
|  |  |  | *Actinobacteria.Tropheryma* |
|  |  |  | *Proteobacteria.Neisseria* |
|  |  |  | *Proteobacteria.Pasteurellaceae* |
|  |  |  | *Fusobacteria.Fusobacerium* |
|  |  |  | *Bacteroidetes.Porphyromonas* |
| BAL | 2 healthy controls | *Firmicutes* | *Proteobacteria.Haemophilus* |
|  |  | *Proteobacteria* | *Firmicutes.Streptococcus* |
|  |  | *Actinobacteria* | *Firmicutes.Lactobacillus* |
|  |  | *Bacteroidetes* | *Firmicutes.Dolosigranulum* |
|  |  | *Fusobacteria* |  |
| BAL | 8 control subjects |  | *Firmicutes.Streptococcus* |
|  |  |  | *Firmicutes.Veillonella* |
|  |  |  | *Bacteroidetes.Prevetella* |
|  |  |  | *Proteobacteria.Haemophilus* |
|  |  |  | *Actinobacteria.Actinomyces* |
|  |  |  | *Actinobacteria.Rothia* |
|  |  |  | *Firmicutes.Gemella* |
|  |  |  | *Firmicutes.Lachnospira* |
| Lung tissue | 4 lung transplant | *Proteobacteria* | *Proteobacteria.Pseudomonas* |
|  |  | *Firmicutes* | *Proteobacteria.Haemophilus* |
|  |  |  | *Proteobacteria.Chryseomonas* |
|  |  |  | *Firmicutes.Streptococcus* |
|  |  |  | *Firmicutes.Staphylococcus* |
|  |  |  | *Proteobacteria.Burkholderia* |
| Lung tissue | ex-plant lung of a transplant patient | *Proteobacteria* | *Proteobacteria.Comomonadaceae* |
|  |  | *Bacteriodetes* | *Bacteriodetes.Flavobacteriaceae* |
|  |  | *Firmicutes* | *Firmicutes.Lactobacillus* |
|  |  | *Actinobacteria* | *ActinobacteriaPropionibacterium* |
|  |  | *Cyanobacteria* | *Proteobacteria.Sphingomonadaceae* |
|  |  |  | *Firmicutes.Staphylococcus* |
| Lung tissue | deceased CF patient | *Proteobacteria* | *Proteobacteria.Pseudomonas* |
|  |  | *Bacteriodetes* | *Firmicutes.Lactobacillus* |
|  |  | *Firmicutes* | *Bacteriodetes.Flavobacteriaceae* |
|  |  | *Actinobacteria* | *Proteobacteria.Sphingomonadaceae* |
|  |  | *Cyanobacteria* |  |
|  |  |  |  |
| Lung tissue | 5 COPD lung | *Proteobacteria* |  |
|  |  | *Firmicutes* |  |
|  |  | *Bacteroidetes* |  |
|  |  | *Actinobacteria* |  |
| Lung tissue | 4 donor lung,healthy | *Proteobacteria* |  |
|  |  | *Bacteroidetes* |  |
|  |  | *Firmicutes* |  |
|  |  | *Actinobacteria* |  |
| Lung tissue | 8 nonsmokers, 8 smokers without COPD, 8 COPD and 8 CF | *Proteobacteria* | *Proteobacteria.Comomonadaceae* |
|  |  | *Bacteroidetes* | *Proteobacteria.Pseudomonas* |
|  |  | *Firmicutes* |  |
|  |  | *Actinobacteria* |  |
|  |  | *Cyanobacteria* |  |

Note: BAL, Bronchoalveolar lavage; COPD, Chronic obstructive pulmonary disease; LUL, left upper lobe; CF, Cystic Fibrosis.

The relative abundance from Bassis *et al.* was calculated from the OTU table the authors sent us. The taxa reported for all studies are ranked from high to low relative abundance.

**References:**

1. Bassis CM, Erb-Downward JR, Dickson RP, Freeman CM, Schmidt TM, Young VB, et al. (2015) Analysis of the Upper Respiratory Tract Microbiotas as the Source of the Lung and Gastric Microbiotas in Healthy Individuals. MBio 6:e00037.

2. Dickson RP, Erb-Downward JR, Freeman CM, Walker N, Scales BS, Beck JM, et al. (2014) Changes in the Lung Microbiome following Lung Transplantation Include the Emergence of Two Distinct Pseudomonas Species with Distinct Clinical Associations. PLoS One 9: e97214.

3. Hilty M, Burke C, Pedro H, Cardenas P, Bush A, Bossley C, et al. (2010) Disordered microbial communities in asthmatic airways. PLoS One 5: e8578.

4. Charlson ES, Bittinger K, Haas AR, Fitzgerald AS, Frank I, Yadav A, et al. (2011) Topographical continuity of bacterial populations in the healthy human respiratory tract. Am J Respir Crit Care Med 184: 957-963.

5. Erb-Downward JR, Thompson DL, Han MK, Freeman CM, McCloskey L, Schmidt LA, et al. (2011) Analysis of the Lung Microbiome in the "Healthy" Smoker and in COPD. PLoS One 6.

6. Pragman AA, Kim HB, Reilly CS, Wendt C, Isaacson RE (2012) The Lung Microbiome in Moderate and Severe Chronic Obstructive Pulmonary Disease. PLoS One 7:e47305.

7. Segal L. N., Alekseyenko A. V., Clemente J. C., Kulkarni R, Wu B, Chen H, et al. (2013) Enrichment of lung microbiome with supraglottic taxa is associated with increased pulmonary inflammation. Microbiome 1:19.

8. Morris A, Beck JM, Schloss PD, Campbell TB, Crothers K, Curtis JL, et al. (2013) Comparison of the Respiratory Microbiome in Healthy Nonsmokers and Smokers. Am J Respir Crit Care Med 187: 1067-1075.

9. Borewicz K, Pragman AA, Kim HB, Hertz M, Wendt C, Isaacson RE (2013) Longitudinal analysis of the lung microbiome in lung transplantation. FEMS Microbiol Lett 339: 57-65.

10. Willner DL, Hugenholtz P, Yerkovich ST, Tan ME, Daly JN, Lachner N, et al. (2013) Reestablishment of Recipient-associated Microbiota in the Lung Allograft Is Linked to Reduced Risk of Bronchiolitis Obliterans Syndrome. Am J Respir Crit Care Med 187: 640-647.

11. Willner D, Haynes MR, Furlan M, Schmieder R, Lim YW, Rainey PB, et al. (2012) Spatial distribution of microbial communities in the cystic fibrosis lung. ISME J 6: 471-474.

12. Sze MA, Dimitriu PA, Suzuki M, McDonough JE, Campbell JD, Brothers JF, et al. (2015) Host Response to the Lung Microbiome in Chronic Obstructive Pulmonary Disease. Am J Respir Crit Care Med 192: 438-445.

13. Sze MA, Dimitriu PA, Hayashi S, Elliott WM, McDonough JE, Gosselink JV, et al. (2012) The Lung Tissue Microbiome in Chronic Obstructive Pulmonary Disease. Am J Respir Crit Care Med 185: 1073-1080.

**Table S9**. The top 10 abundant genera in body sites of nasal, oral, skin, stool, vagina and lung tissues

| **Oral** | **Vagina** | **Stool** | **Skin** | **Nasal** | **Lung tissue** |
| --- | --- | --- | --- | --- | --- |
| *Streptococcus (29)* | *Lactobacillus (85)* | *Bacteroides*  *(48)* | *Propionibacterium(6)* | *Corynebacterium*  *(34)* | *Acinetobacter(12)* |
| *Haemophilus (10)* | *Prevotella*  *(3)* | *Alistipes*  *(5)* | *Staphylococcus (15)* | *Propionibacterium (23)* | *Comamonadaceae.unknown(12)* |
| *Prevotella(10)* | *Gardnerella*  *(3)* | *Faecalibacterium*  *(5)* | *Corynebacterium (5)* | *Staphylococcus*  *(19)* | *Thermus(9)* |
| *Veillonella*  *(7)* | *Atopobium*  *(1)* | *Parabacteroides*  *(4)* | *Anaerococcus*  *(2)* | *Moraxella(4)* | *Pseudomonas(7)* |
| *Neisseria*  *(5)* | *Bifidobacterium (1)* | *Prevotellaceae.*  *Prevotella(3)* | *Streptococcus*  *(2)* | *Carnobacteriaceae.unknown(3)* | *Cloacibacterium(6)* |
| *Actinomyces*  *(5)* | *Shuttleworthia (1)* | *Oscillospira*  *(3)* | *Ralstonia (1)* | *Corynebacteriaceae.unknown(2)* | *Oxalobacteraceae.unknown(3)* |
| *Porphyromonas (4)* | *Lactobacillales. unknown(1)* | *Ruminococcus*  *(3)* | *Streptophyta.*  *unknown(1)* | *Streptococcus(2)* | *Ralstonia(3)* |
| *Fusobacterium (4)* | *Pseudomonas*  *(1)* | *Roseburia(2)* | *Bacteroides(1)* | *Anaerococcus(2)* | *Staphylococcus*  *(2)* |
| *Capnocytophaga (3)* | *Anaerococcus (0.5)* | *Bacteroidales.*  *unknown(2)* | *Neisseriaceae.*  *unknown(1)* | *Peptoniphilus(1)* | *Streptococcus*  *(2)* |
| *Leptotrichia*  *(3)* | *Burkholderia (0.4)* | *Ruminococcaceae.unknown(2)* | *Moraxellaceae.*  *unknown(1)* | *Neisseriaceae.*  *unknown(1)* | *Rizobiales.unknown/Burkholderia/Corynebacterium(2)* |

Note: All the data other than lung tissue are from HMP 16S rRNA (V3-V5 region) phase 1. The average relative abundances (%) are shown in parentheses.
