# Supplementary material for: Characterizing human lung tissue microbiota and its relationship to epidemiological and clinical features
Source: Genome Biol. 2016 Jul 28;17:163. doi: 10.1186/s13059-016-1021-1 (PMC4964003; doi:10.1186/s13059-016-1021-1)

Additional file 2

Figure S1. Map showing number of subjects from the five residential areas in Lombardia, Italy, where study subjects resided (denoted with star).

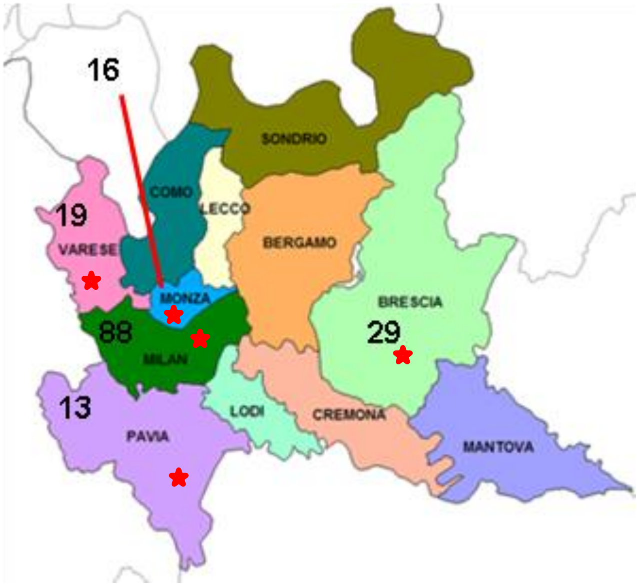

Figure S2. Relative abundance of *Thermi* in HMP phase 1 samples (n=2744). Each circle represents a unique sample or overlapping samples at the same relative abundance. Most samples have no or extreme low relative abundance of *Thermi*.

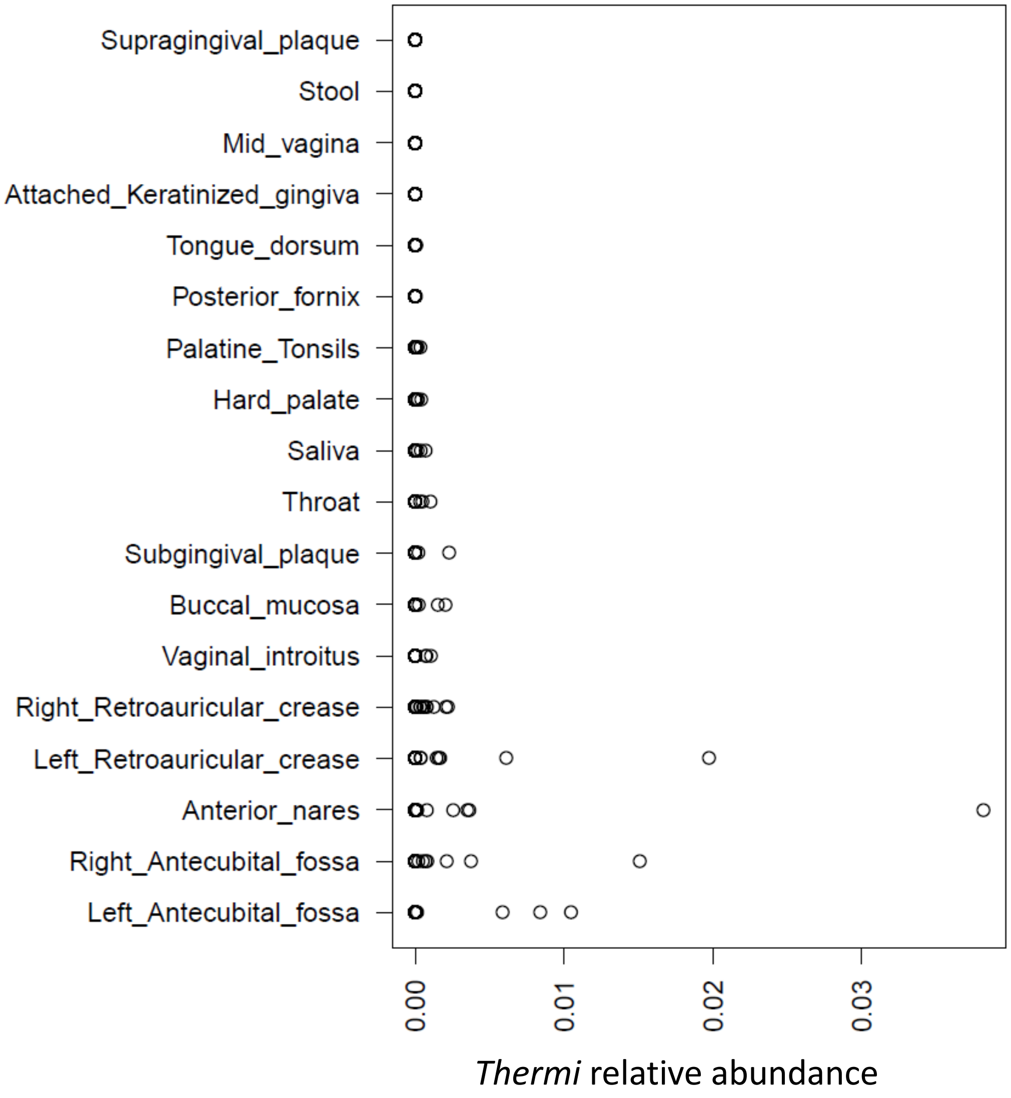

Figure S3. Heatmap of Spearman correlation between phylum-level taxonomic and module-level functional profiles among non-malignant lung tissue samples. Only the top and phylum and modules were shown.

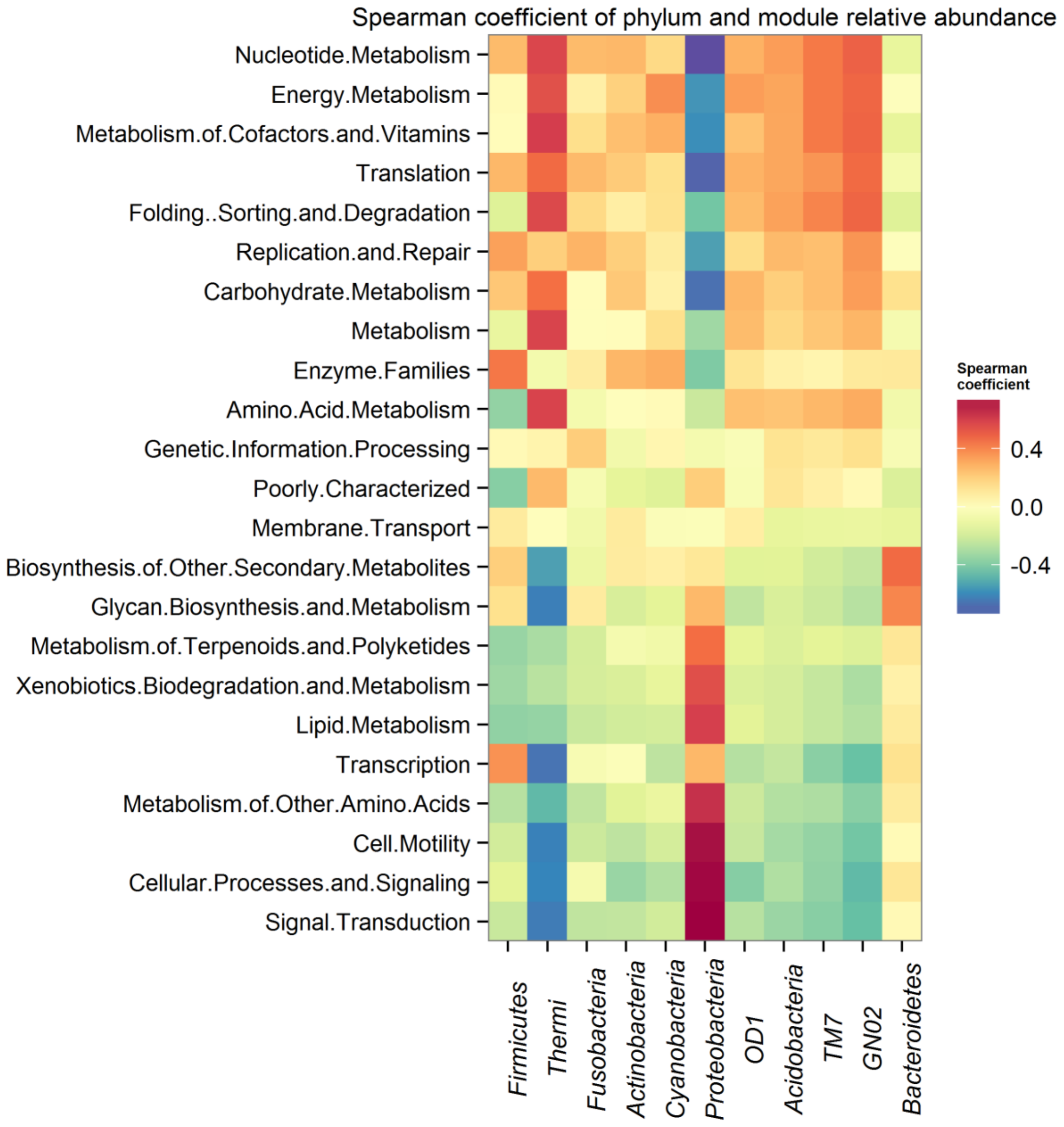

Figure S4. Heatmap showing KEGG modules (marked with star) and pathways that significantly differed in relative abundance by patients' tumor stage (Bonferroni-corrected, P<0.05 according to both Kruskal Wallis test and linear regression model adjusted for history of bronchitis and residential areas (stage IV is the reference)). Row-z scores are the module/pathway relative abundance subtracted from the mean among samples and divided by the standard deviation.

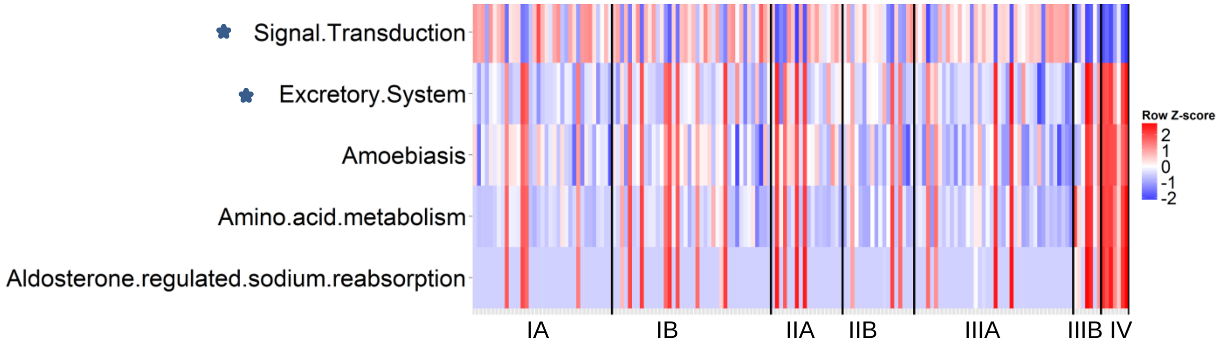

Figure S5. Comparison of mean between- and within-subjects Weighted UniFrac distance. UniFrac distance between-subject in non-malignant tissues (N), tumor tissues (T) and UniFrac distance between paired N and T tissues within-subject (NT) were compared. There is significantly lower mean distance within- than between-subjects; but, no significant difference of mean between-subjects distance comparing non-malignant *vs.* tumor samples.

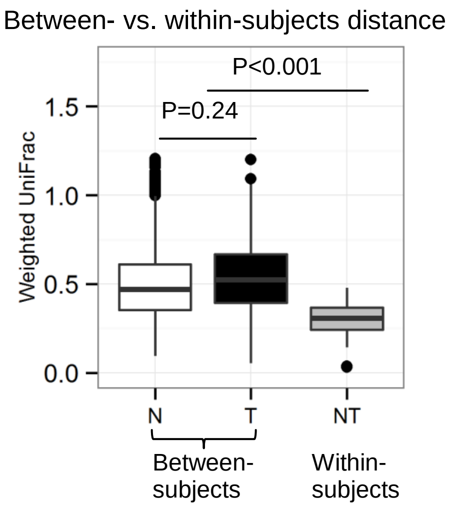

Supplement: Additional file 2: — Supplementary Figures S1 through S5. (PDF 630 kb) [file 13059_2016_1021_MOESM2_ESM.pdf]
